# GeNESiS: gene network evolution simulation software

**DOI:** 10.1186/1471-2105-9-541

**Published:** 2008-12-16

**Authors:** Anton Kratz, Masaru Tomita, Arun Krishnan

**Affiliations:** 1 Institute for Advanced Biosciences, Keio University, 14-1, Baba-Cho, Tsuruoka, Yamagata-ken, 997-0035, Japan

## Abstract

**Background:**

There has been a lot of interest in recent years focusing on the modeling and simulation of Gene Regulatory Networks (GRNs). However, the evolutionary mechanisms that give rise to GRNs in the first place are still largely unknown. In an earlier work, we developed a framework to analyze the effect of objective functions, input types and starting populations on the evolution of GRNs with a specific emphasis on the robustness of evolved GRNs.

**Results:**

In this work, we present a parallel software package, GeNESiS for the modeling and simulation of the evolution of gene regulatory networks (GRNs). The software models the process of gene regulation through a combination of finite-state and stochastic models. The evolution of GRNs is then simulated by means of a genetic algorithm with the network connections represented as binary strings. The software allows users to simulate the evolution under varying selective pressures and starting conditions. We believe that the software provides a way for researchers to understand the evolutionary behavior of populations of GRNs.

**Conclusion:**

We believe that GeNESiS will serve as a useful tool for scientists interested in understanding the evolution of gene regulatory networks under a range of different conditions and selective pressures. Such modeling efforts can lead to a greater understanding of the network characteristics of GRNs.

## Background

While a lot of interest has been focused on the modeling and simulation of Gene Regulatory Networks (GRNs) in recent years, the evolutionary mechanisms that give rise to GRNs in the first place are still largely unknown. There have been efforts at understanding particular aspects of evolution, such as the correlation between development, evolution and robustness or canalization of the network ([1,2]). Studies on the evolution of GRNs have tended to focus on certain a *priori *assumptions about the nature of the evolutionary force such as stabilizing selection ([3-6]) or the use of more abstract ([7]) and analytical ([8]) models. Siegal *et al*. [1] showed that the developmental process constrains the genetic system to produce robustness even in the absence of a selection towards optimum.

In an earlier work [9], we developed a framework to analyze the effect of objective functions, input types and starting populations on the evolution of GRNs with a specific emphasis on the robustness of evolved GRNs. We observed that robustness evolves along with the networks as an emergent property even in the absence of specific selective pressure. During this optimization process towards a more robust system, multiple genotypes evolve which give rise to the same phenotype; this is in accordance with the theoretical view that natural selection operates on phenotypes, thereby accommodating variation in the genotype by fixing those changes that are phenotype-neutral.

In this work, we introduce a parallel software package GeNESiS (Gene Network Evolution SImulation Software) that implements the framework developed in [9] for studying the evolution of GRNs.

## Software

The software GeNESiS, which stands for GEne Network Evolution SImulation Software, is composed essentially of two parts: The front-end graphical user interface (GUI) written in Java and the backend algorithm written in C. The algorithm itself is parallel in nature and has been built using the GNU Scientific Library (GSL) [10] and the parallel genetic algorithm package (PGAPACK) [11]. A slight change was made in the data structure of PGAPACK in order to pass parameters from one subroutine to another. Moreover, since PGAPACK uses the Message Passing Interface (MPI) [12] in order to run parallely across multiple processors, the algorithm requires the presence of some MPI implementation and has been tested using both, MPICH [13] and OpenMPI [14].

### Algorithm

At the basic level, the model consists of a finite-state aspect since the state of the network depends on the binding/unbinding of proteins to the different binding sites in the promoter regions of the different genes. Each protein has binding domains for none or more genes. The effect of a protein binding to the promoter region of a gene can be activation or repression. In addition, a protein can also undergo an activating or a repressing post translational modification (PTM). A similar abstraction can also be made for the RNAP-cofactor complexes. Each RNAP-confactor complex can bind to none or more genes in order to transcribe them. The RNAP-cofactor complexes also evolve by either gaining or losing the ability to bind to and transcribe specific genes.

The gene activity in our model is governed by the number of molecules of the "active" gene (that is one with promoter proteins bound to their promoter regions) as a result of which, the model stays closer to reality where a basal level of gene activity is present and genes are seldom seen to exhibit purely binary state behavior. Additionally, in contrast to the work by Brazma *et al*. [15], time, in our case is discrete. Moreover, the state affects the number of molecules of each species in the system. Additionally, we also model the effect of reversible PTMs. We describe the model in more detail in the following section.

### Model

Our model of gene regulatory networks has been discussed in detail in [9]; here we give only a brief description of the model. Our model attempts to describe the process of gene regulation from the binding of the transcription factor-RNA Polymerase complex to the DNA molecule to the translation of mRNA into the protein product. Every gene is represented by a DNA molecule that is assumed to have one or more sites for the binding of transcription factors and other cofactors. The RNA Polymerase molecule can then bind to the transcription factor-cofactor complex which then breaks down on completion of the reading to form the mRNA molecule which is in turn translated to form the associated protein molecule. The protein molecule can cause both positive and negative regulation of its target gene. However, negative regulation is not independent of the binding order which implies that the molecule can only bind to the promoter region of its target gene in the absence of any other transcription factors. The protein molecule can also undergo post-translational modifications (PTM) which can be both enabling and disabling modifications. Enabling modifications turn the activity of a protein molecule "on" while an inactivating modification turns the activity of the molecule "off".

### Network Evolution

The evolution of the networks is studied by means of a genetic algorithm (GA). A bitstring representation of the different RNAP-cofactor complexes and the protein molecules is concatenated together to form a representation of the entire network. Each such representation of a network is used as an individual chromosome in the genetic algorithm. At the start, a population of solutions is initialized using networks with random connectivities or ones in which all proteins have broad specificities. These correspond to two scenarios: random connectivities corresponding to specificity of DNA-protein interactions while those in which all proteins are connected to each other correspond to the situation whereby any protein can activate any other protein leading to very broad specificities. Once the initial population has been seeded, evolution is allowed to proceed. In each generation, two individuals in the population are chosen at random to mate in order to produce offspring. Individual networks are also subject to mutations while unfit individuals die out, only to be replaced by newer networks. Evolution proceeds under certain pre-defined selective pressures such as maximizing the biomass or through minimizing the number of interactions between proteins or a combination of both selective pressures. Evolution stops when stable networks are obtained. The reader is referred to [9] for more details on the algorithm.

### Graphical User Interface

**Figure 1:**
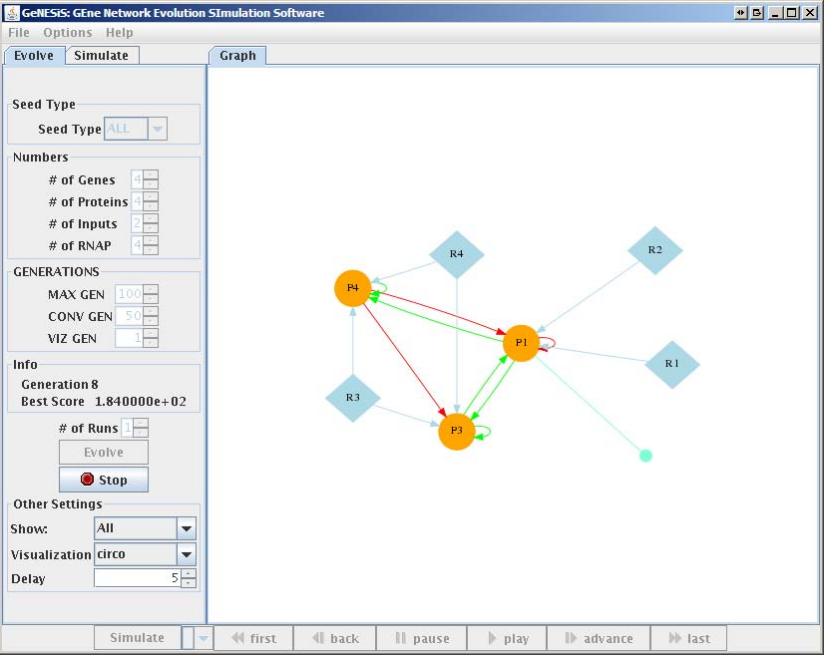
**Evolution Window**.

**Figure 2:**
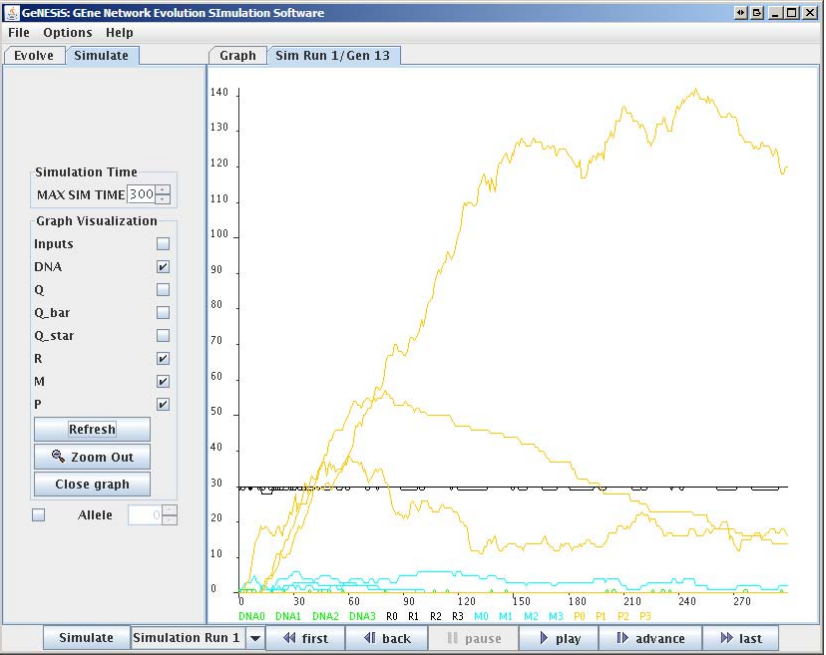
**Simulation Window**.

The GUI for GeNESiS has been written in Java and contains two main canvases as shown in Figures 1 and 2. The "Evolve" tab is used for the evolution of a given network while the "Simulate" tab is used to simulate a particular GRN with the desired parameters. The two essential canvases and their associated functionalities will be discussed in more detail below.

#### GRN Evolution

The main window has two panels on the left and a single canvas on the right, when it is first launched. This canvas is linked to the tab *Evolve *on the left. When the program is launched, the panels as well as the canvas are greyed out. The user can either open a saved project or start a new project. Starting a new project essentially results in the creation of a new directory that will hold the files resulting from one or more runs of the network evolution. For each run, a subdirectory is created within the main project directory (*simrun1*, *simrun2*,...,etc.). Inside each subdirectory are a number of different files that are generated during the evolution run. There are four main types of files generated as given below:

• **GPIR**: This file contains the number of genes (G), proteins (P), inputs (I) and RNAP-cofactor complexes (R) used during the run, in a single column.

• **LOG**: This contains the logged output from the run. The log interval can be set using the *Parameters *tab (see Section on parameters, below). Each line contains the generation number, the best solution for that particular generation, the fitness function score for that solution and the values of the protein levels.

• **LOG.pop**: This file contains, for each generation, the list of all individuals in the population and their fitness function values.

• *.dot: These files contain the network realization in dot format. For each generation, three different *.dot files are created; one each for the case where all the species are shown, only proteins are shown and only RNAP-cofactor complexes along with the genes are shown.

Opening an aleady existing project simply implies opening the main project directory containing the many simulation runs.

The *Evolve *tab contains some of the main parameters required for the evolution of the network such as the number of genes, proteins, inputs and RNAP-cofactor complexes, the number of generations to evolve the network as well as the minimum number of generations for the fitness function to be unchanged before converging. The panel also has a pull down button to change the seed type for the network as well as buttons to start and stop the evolution. There are also graphing options available that allow the user to select the species that will be displayed. GeNESiS uses the GraphViz [16] package to draw the networks and there is a further option to select the particular GraphViz program to use, namely, *dot, circo *or *twopi*, which result in different network layouts.

When the EVOLVE button is pressed, the network evolution algorithm executes in the background and the value of the fitness function for the best network for each generation, along with the generation number is shown in the panel. In addition, the best network for each generation is automatically drawn on the graphing canvas on the right using one of the three different GraphViz programs as set in the panel. Once the evolution has converged or has been manually stopped, a user can follow the evolutionary path by making use of the "movie" buttons at the bottom of the canvas. A number of different runs can be carried out and the results viewed at any given point in time.

#### GRN Simulation

GeNESiS also allows the user to simulate a given GRN. The network that is simulated is the one currently appearing on the graphing canvas. The results from the simulation of one such example network, are shown in Figure 2. The simulation canvas is controlled by the *Simulate *pane to the left. This pane contains options that control the display of the different simulation curves. A zoom functionality has also been provided. This panel also allows the user to mutate the network (by either mutating one of the connections or the PTM or RNAP-cofactor complex binding ability). However, at any given time, only one mutation can be carried out for the base network.

#### Parameters

**Figure 3:**
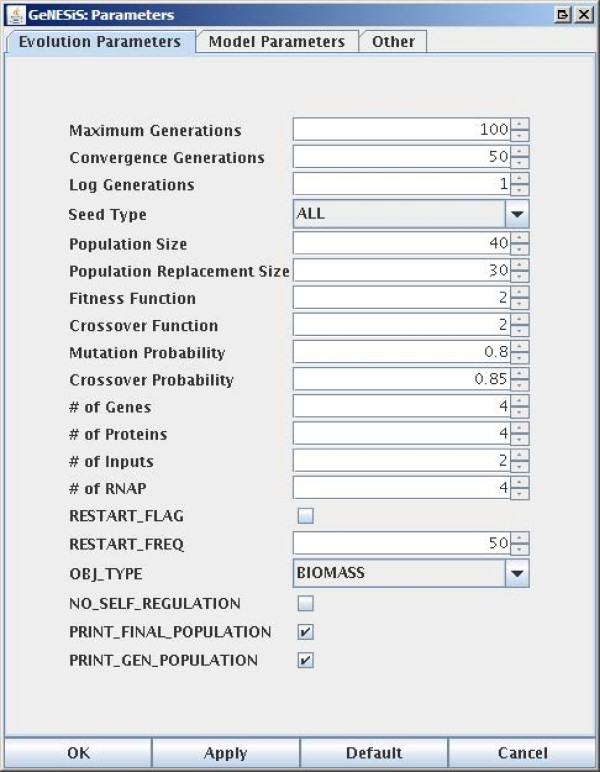
**The parameters panes: Evolution Parameters**.

**Figure 4:**
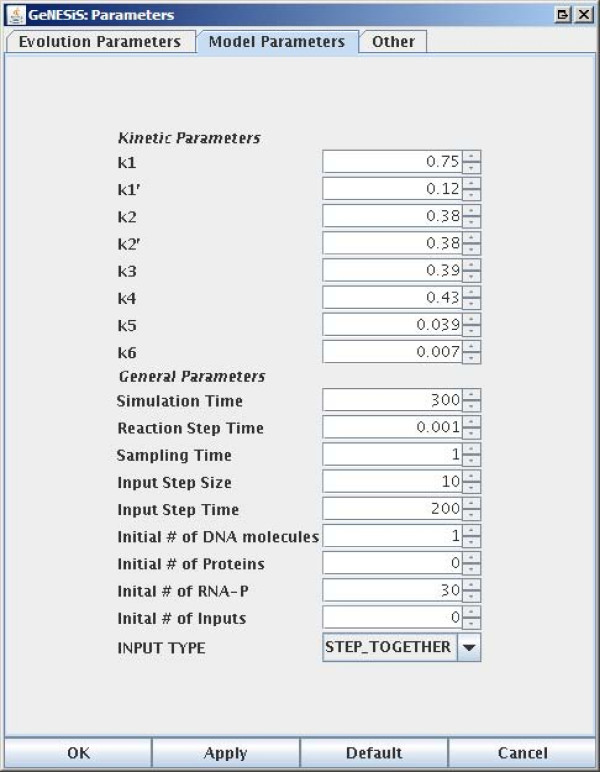
**The parameters panes: Simulation Parameters**.

**Figure 5:**
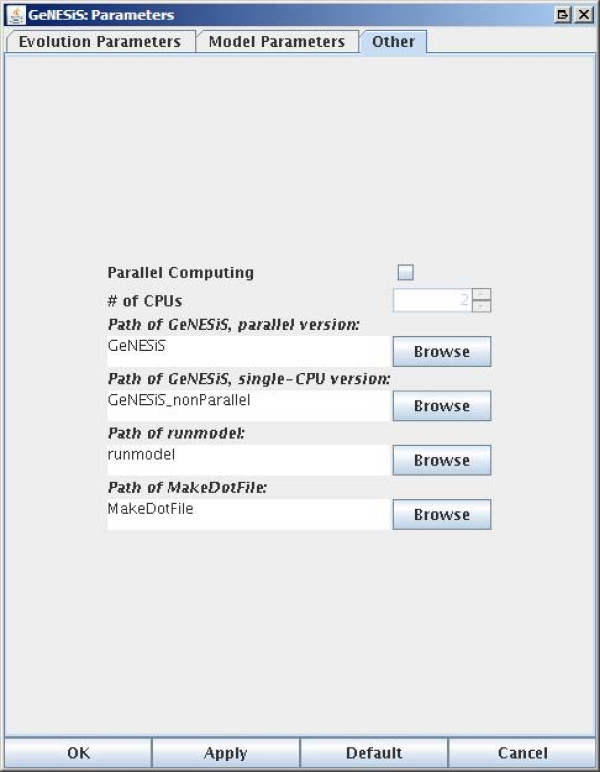
**The parameters panes: Other Parameters**.

GeNESiS has a number of parameters that can be set, both for the evolution of the networks as well as for simulating a given network. The three different tabs on the *Parameters *pane corresponding to the *Evolution*, *Model *and *Other *parameters are shown in Figures 3, 4 and 5 respectively.

#### Evolution Parameters

The *Evolution Parameters *tab contains parameters that are typically used by genetic algorithms such as the maximum number of generations, the maximum number of generations to converge to a solution (that is, the maximum number of generations for a solution to not change in order to assume convergence), the size of the population, the population replacement size and the mutation and crossover probabilities. The users are directed to [11] for more details about the parameters used in the genetic algorithm.

There are four different objective functions that can be used for evolution, called, *Biomass, Robustness, Total *and *Biomass_plus_min_links*. *Biomass *tends to maximize the difference between those proteins which don't have external inputs and those which do; *Robustness *tries to explicitly maximize the robustness of the system by picking the solution which undergoes the least change to all point mutations of the bitstring; *Total *tries to maximize the sum of all the proteins in the system and *Biomass_plus_min_links *tries to maximize the biomass as before, while at the same time minimizing the total number of connections in the network.

The *Evolution Parameters *tab also has options to set the numbers of genes, proteins, inputs and RNAP-cofactor complexes in the system and these are linked to their counterparts on the *Evolve *pane. There are also options here to print the final population as well as the population every generation.

#### Model Parameters

This tab contains all the parameters required to simulate the model of a given gene regulatory network. It contains the kinetic parameters (in effect these are actually the reaction probabilities) for all the reactions as well as general parameters such as the simulation time, the reaction step time and the sampling time. This tab also contains the initial numbers of the DNA molecule, proteins, RNAP-cofactor complexes as well as the inputs. The input step size as well as the input step time can be set here. These inputs are basically proteins which are external to the network, but still have a role in the activation of transcription or in the repression of some of the proteins of the network.

#### Other Parameters

This tab contains miscellaneous parameters such as the paths for the different executables as well as a checkbox for the use of multiple CPUs. The software package contains a detailed user manual that describes the software in greater detail along with examples.

## Conclusion

Our earlier study [9] on GRN evolution using GeNESiS enabled us to conclude that it can serve as an important tool for analyzing the evolution of GRNs. The ability to study GRN evolution under different selective pressures and starting conditions is an inherent strength of the GeNESiS framework. We foresee GeNESiS being used for large-scale simulation of GRN evolution. Future enhancements to GeNESiS would include the ability to make use of the grid framework to launch massively-parallel simulations with hundreds of genes.

## Availability and requirements

• *Availability*: The software is freely downloadable from .

• *Programming Language*: The core algorithm is written in C while the GUI is written in Java.

• *Dependencies*: GNU Scientific Library, PGAPACK, GraphViz, OpenMPI/MPICH

• *Platforms*: Linux/Unix-based

## Authors' contributions

AKrishnan created the main algorithm and wrote the paper. AKratz designed and developed the software and also helped in the writing of the paper. MT was in charge of the overall project. All authors read and approved the final manuscript.

## References

[B1] Siegal ML, Bergman A (2002). Waddington's canalization revisited: Developmental stability and evolution. Proc Natl Acad Sci USA.

[B2] Bergman A, Siegal ML (2003). Evolutionary capacitance as a general feature of complex gene networks. Nature.

[B3] Gavrilets S, Hastings A (1994). A quantitative-genetic model for selection on developmental noise. Evolution.

[B4] Wagner A (1996). Does evolutionary plasticity evolve. Evolution.

[B5] Wagner GP, Booth G, Bagheri-Chaichian H (1997). A population genetic theory of canalization. Evolution.

[B6] Ancel LW, Fontana W (2000). Plasticity, evolvability and modularity in rna. J Exp Zool.

[B7] Rice SH (1998). The evolution of canalization and the breaking of von baer's laws: Modeling the evolution of development with epistasis. Evolution.

[B8] Eshel I, Matessi C (1998). Canalization, genetic assimilation and preadaptation: A quantitative genetic model. Genetics.

[B9] Krishnan A, Giuliani A, Tomita M (2008). Evolution of gene regulatory networks: Robustness as an emergent property of evolution. Physica A.

[B10] Pierce R (1996). The gnu scientific software library. http://www.gnu.org/software/gsl/.

[B11] Levine D (1996). Users guide to the pgapack parallel genetic algorithm library. ftp://info.mcs.anl.gov/pub/pgapack/pgapack.tar.Z.

[B12] Mpi Message passing interface. http://www-unix.mcs.anl.gov/mpi/.

[B13] Gropp W, Lusk E, Doss N, Skjellum A (1996). A high-performance, portable implementation of the MPI message passing interface standard. Parallel Computing.

[B14] Gabriel E, Fagg GE, Bosilca G, Angskun T, Dongarra JJ (2004). Open mpi: Goals, concept, and design of a next generation mpi implementation. 11th European PVM/MPI Users' Group Meeting, Budapest, Hungary.

[B15] Brazma A, Schlitt T (2003). Reverse engineering of gene regulatory networks: a finite state linear model. Genome Biology.

[B16] Gansner ER, Koutsofios E, North SC, Vo KP (1993). A technique for drawing directed graphs. Software Engineering.

